# The demonstration of a theory-based approach to the design of localized patient safety interventions

**DOI:** 10.1186/1748-5908-8-123

**Published:** 2013-10-16

**Authors:** Natalie Taylor, Rebecca Lawton, Beverley Slater, Robbie Foy

**Affiliations:** 1 Australian Institute of Health Innovation, Faculty of Medicine, Level 1, AGSM Building, University of New South Wales, Sydney, New South Wales, Australia; 2 Institute of Psychological Sciences, University of Leeds, Leeds, Lupton Place, Leeds, UK; 3 Bradford Institute for Health Research, Bradford Royal Infirmary, Duckworth Lane, Bradford, UK; 4 Leeds Institute of Health Sciences, Charles Thackrah Building, University of Leeds, 101 Clarendon Road, Leeds, UK

**Keywords:** Behavior change, Theoretical domains framework, Intervention, Implementation, Patient safety

## Abstract

**Background:**

There is evidence of unsafe care in healthcare systems globally. Interventions to implement recommended practice often have modest and variable effects. Ideally, selecting and adapting interventions according to local contexts should enhance effects. However, the means by which this can happen is seldom systematic, based on theory, or made transparent. This work aimed to demonstrate the applicability, feasibility, and acceptability of a theoretical domains framework implementation (TDFI) approach for co-designing patient safety interventions.

**Methods:**

We worked with three hospitals to support the implementation of evidence-based guidance to reduce the risk of feeding into misplaced nasogastric feeding tubes. Our stepped process, informed by the TDF and key principles from implementation literature, entailed: involving stakeholders; identifying target behaviors; identifying local factors (barriers and levers) affecting behavior change using a TDF-based questionnaire; working with stakeholders to generate specific local strategies to address key barriers; and supporting stakeholders to implement strategies. Exit interviews and audit data collection were undertaken to assess the feasibility and acceptability of this approach.

**Results:**

Following audit and discussion, implementation teams for each Trust identified the process of checking the positioning of nasogastric tubes prior to feeding as the key behavior to target. Questionnaire results indicated differences in key barriers between organizations. Focus groups generated innovative, generalizable, and adaptable strategies for overcoming barriers, such as awareness events, screensavers, equipment modifications, and interactive learning resources. Exit interviews identified themes relating to the benefits, challenges, and sustainability of this approach. Time trend audit data were collected for 301 patients over an 18-month period for one Trust, suggesting clinically significant improved use of pH and documentation of practice following the intervention.

**Conclusions:**

The TDF is a feasible and acceptable framework to guide the implementation of patient safety interventions. The stepped TDFI approach engages healthcare professionals and facilitates contextualization in identifying the target behavior, eliciting local barriers, and selecting strategies to address those barriers. This approach may be of use to implementation teams and policy makers, although our promising findings confirm the need for a more rigorous evaluation; a balanced block evaluation is currently underway.

## Background

Patient safety is a global priority. An estimated 3.7% to 17.7% of patients are inadvertently harmed as a result of hospital care [1-3]. Some harm is caused by healthcare professional error arising from factors such as poor system and equipment design, and high workload [4,5]. Other harm results from deviations from guidelines and policies; only between 50 and 70% of patients receive recommended care [6,7]. Interventions to change professional behavior have modest and variable effects [8]. This variability is problematic because it limits the ability to predict with any confidence whether an intervention will work for a given problem and context [9]. The reported modest effects may be the result of problems with the ways by which interventions are selected. First, selection may be based more upon habit and disciplinary perspective than an explicit rationale that takes targeted behavior and context into account [10]. Second, the theory underpinning healthcare professional behavior change interventions is seldom explicated, thereby limiting the ability to generalize from one context to another [11]. Third, standardized 'top down’ interventions may lack flexibility to respond to local barriers and circumstances [12,13].

Behavior change occurs within complex social and environmental systems that demonstrate local variations [14]. It could be argued then that interventions to improve patient safety will be most effective when developed by those with local 'expertise’ and tacit knowledge [15-17], but which take account of evidence and external expertise. This paper describes how we co-designed interventions with local stakeholders, guided by both behavior change (impact) theory and implementation (process) theory [18].

We selected the theoretical domains framework (TDF) of behavior change [19] as the 'impact’ theoretical approach for this work. The TDF was developed to rationalize and reconceptualize the theoretical constructs from multiple psychological and organizational theories of behavior and behavior change using an expert consensus and validation process. The resulting framework includes the description of the nature of the behavior to be targeted and eleven domains of behavior change: knowledge, skills, social/professional role and identity, beliefs about capabilities, beliefs about consequences, motivation and goals, memory attention and decision processes, environmental context and resources, social influences, emotion, and behavioral regulation. The framework has been used to understand barriers and levers to change in a range of contexts c.f, [20-26]. We also drew upon principles derived from implementation ('process’) theories [19], which provide insights into the necessary conditions for optimizing adoption and spread of interventions in practice (Table 1). This combined approach will be referred to as the Theoretical Domains Framework Implementation (TDFI) approach.

**Table 1:** Key implementation principles and examples of supporting literature

**Implementation principles**	**Supporting literature**
1. The need for management approval and ongoing support	[17,27]
2. The need for commitment among members of the target group	[18,28]
3. Use of boundary spanners	[29-31]
4. Mapping of guidelines onto local problems	[32-36]
5. Adopting the perspective of the target group	[28,37]
6. Acknowledging the complexity of changing behavior in practice	[37,38]
7. A monitoring plan	[18]
8. A flexible approach that is driven by local context	[37,39]
9. Co-production and design to combine theoretical and contextual expertise	[38]
10. Incorporation into established structures	[38-40]

The TDF has been used for designing interventions to change clinician behavior in primary care [41], but not in an acute setting. We describe how the TDF was operationalized using co-production and implementation principles, and applied to three UK hospitals to improve the implementation of a patient safety guideline promoting safe nasogastric feeding. We addressed two questions: How important is local context in identifying barriers and appropriate interventions to implement safety guidance? How feasible and acceptable is the TDFI approach for implementing patient safety guidance?

## Methods

### Context

Between April 2011 and September 2012 the Yorkshire and Humber Health Innovation and Education Cluster (HIEC) Patient Safety Theme operationalized the TDF to implement National Patient Safety Agency (NPSA) alerts (evidence-based guidelines) in four hospital Trusts (five hospitals as part of a service evaluation). Three of these hospitals (referred to as H1, H2, and H3) chose to work on an alert released in March 2011 that focussed on 'reducing the harm caused by misplaced nasogastric (NG) feeding tubes’ [42]. Misplacement of NG tubes is not uncommon and can have serious consequences; between 2005 and 2011, there were 21 deaths and 79 cases of harm in the UK due to feeding into the lungs. Although there is no completely reliable method for checking tube placement, the guideline recommends that the first line method for confirming tube position should be to check the pH of the aspirate from the stomach. If the pH is >5.5, or obtaining an aspirate is not possible, it is only then appropriate to request an X-ray to check the tube position. The position of the NG tube is not always clear from the X-ray and therefore the risk of misinterpretation is high.

Consultation with the local NHS ethics committee indicated that ethical approval was not required for this work as this project was a service evaluation focusing on NHS staff and data were collected by the teams as part of the implementation process. The exception to this was the exit interviews conducted with staff, for which ethical approval is no longer required.

### Implementation teams

The HIEC team supported H1, H2, and H3 to form clinically led, multidisciplinary implementation teams focusing on the NPSA alert.

### Implementation tools

#### Audit tool

To understand the nature of the target behavior, the HIEC team worked with implementation teams to co-design a tool for auditing notes of patients who had received an NG tube for the purposes of identifying to what extent guideline recommendations were being followed, and to elucidate those behaviors that might be targeted to increase compliance.

### Influences on patient safety behaviors questionnaire

We used the validated Influences on Patient Safety Behaviors Questionnaire IPSBQ; [43] to assess barriers to the target behavior using 11 subscales based on the TDF [19]. Example items include: 'I am confident I can… do X target behavior’ (beliefs about capabilities); 'There is not a good enough system in place for me to…do X target behavior’ (environmental context and resources). Participants rated their level of agreement with each statement on a 5-point likert scale (1 = strongly agree; 5 = strongly disagree). Items were both positively and negatively phrased to counter response set bias. After recoding negatively phrased items, a higher mean score indicates a stronger barrier to behavior change.

### Focus group schedule

Developed to understand the key barriers identified in the IPSBQ, and guide staff generation of intervention strategies (Additional file 1). It contained prompts for the focus group lead to elicit discussion about key barriers, and worksheets for generating ideas for intervention strategies [44].

### Feasibility and acceptability assessment tools

#### Time trend audit tool

A shortened version of the implementation audit tool was used to assess the first line method used to check NG tube position as part of an 18 month long audit in H1.

### Implementation interview schedule

An interview schedule was designed to assess the feasibility and acceptability of the implementation process (Additional file 2).

### Reflective log

The lead researcher (NT) kept a reflective log throughout each phase of the implementation process, describing details and consequences of key challenges faced and solutions generated.

### Implementation procedures

A six-step TDFI approach was tested, each step incorporating one or more of the implementation principles described in Table 1. Steps were: forming an implementation team; defining a locally relevant target behavior; understanding barriers to performing the target behavior; devising intervention strategies to address identified barriers; intervention implementation; and evaluation. The implementation principles for each step are summarized in Additional file 3. Below we focus on the use of the TDF within the implementation process.

### The TDFI approach

Once implementation teams had been established (step one), a locally relevant target behavior was identified (step two) through discussion about local practice and assessment of audit data.

To understand the barriers to performing the target behavior (step three), with support from the implementation team, all staff involved in the target behavior (*e.g.*, doctors, nurses, dieticians, etc.) were invited via email and/or in person to complete the IPSBQ, either online or in paper format. Data were manually entered into a spreadsheet and negatively phrased items were reverse scored. Mean domain scores were calculated for each hospital and an 11 (barrier type) × 3 (hospital) MANOVA was computed to assess differences in the barriers to implementation across the Trusts.

Following analysis of IPSBQ data, focus groups were held at each hospital with multi-disciplinary staff groups from a range of wards and departments. In part one, groups were asked to consider and discuss the 11 barriers relating to the target behavior, then presented with the top four barriers found from the questionnaire data analysis, and (based on these data and their own experiences) asked to come to a consensus about the most influential barriers within their organization. In part two, to devise intervention strategies to address identified barriers (step four), focus group members discussed ideas for intervention strategies that they envisaged would be effective in addressing the most prominent barriers and achieving the target behavior. The generation of the ideas by each group was guided by the project team’s knowledge of the current literature [44-46]. Participants were provided with information about which behavior change techniques (BCTs) had been suggested as effective in addressing each type of barrier. For example, evidence suggests that appropriate BCTs to address 'lack of skill’ include 'modelling or demonstrating the behavior to individuals,’ or for individuals to 'rehearse the relevant skills.’ However, if the barrier related to 'the influence of others,’ appropriate techniques might include 'social processes of pressure, encouragement, or support’ [44].

Focus group data were thematically analyzed using a deductive approach [47]. Each transcript was thoroughly reviewed before extracts of text were themed according to barriers representing the pre-determined TDF domains. The key barriers emerging from the focus groups were cross referenced with those identified by the IPSBQ. Overlap and discrepancies for the top four key barriers were noted. Next, suggested intervention strategies (*e.g.*, changes to a system, improving a protocol, using a screensaver) were matched to specific barriers identified, then mapped against BCTs [45,48].

Once senior management granted permission in each Trust, teams were supported to implement the interventions in their organization (step five), and re-audit case notes to assess change in practice (step six).

### Feasibility and acceptability procedure

#### Time trend audit

To review improvement in the targeted behavior, audit data was collected at H1 for all patients who received an NG tube between January 2011 and June 2012. Given that the extent to which practice was being recorded changed over this time period, we could not perform a formal time series analysis on this data [49]. However, criteria suggested by Perla, Provost, and Murray [40] were used to detect 'signals’ within the data, which can indicate if a process is demonstrating non-random patterns.

### Exit interviews

Implementation team members who had been involved in the project from the beginning (*n* = 5) were approached by an independent interviewer and asked if they would participate in a short telephone interview to discuss their experiences of the process. Inductive thematic analysis was undertaken to identify key emerging themes relating to the acceptability and feasibility of the TDFI approach.

### Reflective log

A reflective log was recorded to capture the challenges presented and solutions generated throughout this process in order to provide an insight into the feasibility and acceptability of the TDFI approach. The solutions were themed according to the ten implementation principles stated in the introduction and mapped against each implementation step.

## Results

### Target behavior

Following discussions with the implementation teams and ward staff, and assessment of audit results (Table 2), each hospital decided that the target behavior for change would be for staff to check pH first line.

**Table 2:** Nasogastric tube audit results from each hospital

**Audit information**	**Hospital 1**	**Hospital 2**	**Hospital 3**
Number of sets of notes audited	49	43	44
First line method used to check NG tube position			
pH of aspirate from patient’s stomach	18%	11%	14%
Patient sent for X-ray	49%	76%	40%
Information not documented	29%	9%	9%
N/A (placed in radiology)	4%	4%	37%

### Key barriers to performing the target behavior

#### IPSBQ data

Questionnaire data were collected from 227 staff members across the three hospitals. Recruitment and sample details, and reliability and validity properties of the IPSBQ are reported elsewhere [43]. Combined mean domain scores (assessing barriers) were calculated separately for each hospital (Table 3). High mean scores represent stronger barriers. Generally, the mean reported barrier scores were low, despite audit results demonstrating poor compliance with recommendations. The strongest barrier to performing the target behavior (checking pH first line) across H1 and H2 was 'social influences’ (the influence of others on the behavior), and for H3 was 'skills (having the necessary training and skills to perform the behavior). There were differences across sites with regards to other reported barriers. For example, the second strongest barrier reported by H1 was 'environmental context and resources’ (systems and resources associated with the behavior), by H2 was 'emotion’ (fears and anxieties associated with the behavior), and by H3 was 'social influences.’

**Table 3:** Descriptive statistics and MANOVA results for barriers across each hospital

**Barrier**	**Mean (SD) H1**	**Mean (SD) H2**	**Mean (SD) H3**	**Mean (SD) all hospitals**
	***n = 99***	***n =105***	***n =23***	***n = 227***
Knowledge	2.02 (0.70)	2.33 (0.75)	2.08 (0.76)	2.17 (0.74)**
Skills	2.37 (0.79)	2.64 (0.72)	2.74 (0.87)	2.53 (0.78)**
Social and professional identity	2.04 (0.73)	1.96 (0.64)	2.16 (0.79)	2.01 (0.69)
Beliefs about capabilities	2.44 (0.77)	2.55 (0.83)	2.52 (0.97)	2.50 (0.81)
Beliefs about consequences	2.35 (0.70)	2.38 (0.70)	2.39 (0.48)	2.37 (0.68)
Motivation and goals	2.40 (0.66)	2.40 (0.60)	2.65 (0.69)	2.42 (0.64)
Cognitive processes, memory and decision making	2.36 (0.68)	2.47 (0.74)	2.19 (0.67)	2.39 (0.71)
Environmental context and resources	2.55 (0.85)	2.69 (0.69)	2.68 (0.62	2.63 (0.76)
Social influences	2.84 (0.76)	2.89 (0.73)	2.71 (0.75)	2.85 (0.74)
Emotion	2.41 (0.65)	2.75 (0.55)	2.35 (0.62)	2.56 (0.63)*
Action Planning	2.32 (0.66)	2.38 (0.62)	2.42 (0.54)	2.36 (0.63)

*p < 0.01*;* **p < 0.05 for MANOVA results of differences between reported barriers in each hospital.

NB: Mean scores for individual hospitals were computed following initial reliability analysis on each dataset; mean scores were taken from analysis for the combined data set following missing values analysis (prior to confirmatory factor analysis, results of which are reported in [43].

An 11 (barrier type) × 3 (hospital) MANOVA indicated that there was a main effect of hospital on the strength of barrier types reported *F* (2, 224) = 2.88, *p* <0.001, *d* = 0.77. Between subjects effects demonstrated significant differences between hospitals for three of the 11 barrier types: 'knowledge’ *F* (2, 224) = 4.59, *p* <0.05, *d* = 0.40, 'skills’ *F* (2, 224) = 4.17, *p* <0.05, *d* = 0.39, and 'emotion’ *F* (2, 224) = 9.79, *p* <0.001, *d* = 0.59. Further inspection of pairwise comparisons indicated that significant differences were found between H1 and H2 for 'knowledge’ (mean diff = 0.304, *p* <0.05) and 'skills’ (mean diff = 0.274, *p* <0.05), and between H1 and H2 (mean diff = 0.343, *p* <0.001), and H2 and H3 (mean diff = 0.402, *p* <0.05) for 'emotion.’ No significant differences between organizations were found for the other reported barrier types.

### Focus group data

Details of focus group participants are provided in Additional file 1. The introduction and explanation of the barriers to using pH as the first line method to checking NG tube position from the IPSBQ prompted focus group participants to reflect on some of their own experiences relating to this target behavior. For the most part, the top four barriers identified by the IPSBQ emerged from the focus group data (Table 4). For H3, there was one discrepancy for the fourth strongest barrier. Table 5 provides examples of the responses of staff (key barriers and intervention suggestions) mapped against TDF domains.

**Table 4:** Comparison of key barriers identified by the IPSBQ and focus groups

**Hospital**	**IPSBQ top 4 barriers**	**Focus group consensus top 4 barriers**
1	Social influences	Social influences
	Environmental context and resources	Environmental context and resources
	Beliefs about capabilities	Beliefs about capabilities
	Emotion	Emotion
2	Social influences	Social influences
	Emotion	Emotion
	Environmental context and resources	Environmental context and resources
	Skills	Skills
3	Skills	Skills
	Social influences	Social influences
	Environmental context and resources	Environmental context and resources
	*Motivation and goals*	*Emotion*

**Table 5:** TDF domains, mapped against summary of barrier and example quotes

**TDF domain**	**Example of barrier**	**Quote representing barrier**	**Quote representing intervention suggestions**
Social influences	Feeling pressured into doing an X-ray first	'If my boss told me to do one it would be very difficult for me to, depending on which the boss was, generally you’d be like no but don’t you know that local guidelines are…they’d be like I said get a chest x-ray, you’d be like oh alright’ (Junior doctor, H1).	'Well you’ve got to bring the consultants on board…I think it needs a big cascade…we could have it as a screen saver (Junior doctor, H2).
			'If at one point during a couple of weeks all the screen savers had something about NG tubes, a load of posters and then there was sort of a couple of meetings or something…what you want to do its just to raise awareness and people will actually think about it a lot more and that’s what you can hope for’ (Consultant, H2).
		'They [nurses] always justified it with 'we’d rather get an x-ray, we’re told not to feed without an x-ray.’ I pushed a couple of times, when I was very confident, when it had gone down very easily it was very acidic…but quite frequently they’d still send for an x-ray or they’d get someone else to request the x-ray, you know, they were adamant they wanted the x-rays and wanted them reported’ (Junior doctor, H2).	
Skills	Working with staff who lacked the correct skills or necessary training	**'**What I’ve identified……is that I get newly qualified staff nurses coming through who have never been taught this as a method of checking, don't know how to check it’ (Dietician, H3).	'The Trust should to do teachings about the use of ph paper vs x-ray, rather than just bombard staff with information’ (Junior doctor, H1).
			'Specific training should be targeted to relevant groups rather than lots of different types of mandatory training’ (Operation Department Practitioner, H1).
		'I think a lot of it is to do with the training, I was talking to a few junior doctors in respiratory and a lot of them haven’t even heard about the training package on the website, but they’re putting tubes down.’ (Nurse, H2).	
			**'**I think the (e-learning) package would be good…If its interactive people are more likely to do it’ (Junior doctor, H2).
Beliefs about capabilities	Low levels of confidence for checking the pH level	'…people just aren’t checking the aspirate and we almost need to get them to just check and then even if they are unsure, fine send for an x-ray, but if you see that those then correlate and you see that more and more often, then your confidence might increase.’ (Junior Doctor, H2).	'Another way to bring it across would be to have a teaching event or something’ (Nurse, H2).
		'I think confidence would increase if staff knew they were learning the correct skills’ (Senior nurse, H1).	
Environmental context and resources	The lack of resources, such as pH paper or lack of forms for documentation, often leaves doctors with no choice but to send for an x-ray in order to make the decision to feed	'We’re still having problems getting strips; was looking for some this morning and there weren’t any in the cupboard so I had to pinch some from another patient’ (Junior doctor, H1).	'Can you get it in the packs? Like the IV catheter packs? You’ve got all the stuff for your aseptic technique…maybe you need a similar NG pack so people don’t forget that here’s your 20 ml syringe that you aspirate with; here’s your litmus paper…’ (H2: junior doctor).
		'I believe that some of the problems come about where to document it…so it's getting the pH and where do you document that…’ (Nurse, H3).	
			'Someone developed these catheter packs that have all the equipment you need. Could there not be an NG tubes pack with all the necessary equipment for everyone to follow in a specific order?’ (H1, junior doctor).
Emotion	Certain staff do not want to rely on the pH value and feel more comfortable if they have sent for an x-ray	'I think the nurses are still quite anxious because it’s so big even now I think they’re still anxious about pH and they just want to know that it’s in the right place’ (Junior doctor, H1).	'We could provide junior doctors with information about the use of x-rays and potential problems these cause’ (Junior doctor, H1).
		I think there is very much a fear isn’t there, once you can’t get that thing back it’s, you know… (Nurse, H2).	
			'I think also the 50% of the deaths that occurred were from misinterpretation of x-rays. I think if you told F1’s that, even that on a poster, I think, you know, if you caught that out of the side of your eye as an F1…’ (Junior doctor, H2);
			'I would look at it as I went past if it was an x-ray…because a lot of questions that come from the requirement for x-rays are not seen by the people who interpret the x-rays so I think that (a poster with information regarding misinterpretation of X-rays) would be really good’ (Junior doctor, H2).

### Devising intervention strategies to address identified barriers

#### Focus group data

Examples of intervention suggestions matched to quotes representing barriers from specific TDF domains are presented in Table 5.

### Step five: Intervention implementation

Strategies authorized, developed, and implemented across the three Trusts are presented in Table 6. These strategies are mapped alongside the main identified barriers for each Trust, as well as the BCTs that have been suggested as appropriate for addressing specific barriers [44].

**Table 6:** Key barriers, implemented interventions, and associated behavior change techniques

**Key barriers**	**Implemented interventions**	**Behavior change techniques**
Social influences	• Screensaver implemented with key messages targeting social influences	Credible source; Information about health consequences, and social/ environmental consequences; Prompts/cues; Social processes of encouragement, pressure, and support; Provide information about others approval
	• Awareness day/ awareness week*	
Emotion	• Screensaver implemented with key messages targeting emotion	Anticipated regret; Salience of consequences; Framing/reframing
	• Posters implemented with key messages targeting emotion	
Environmental context and resources	• New documentation released (care pathway for NG tubes)	Prompts/cues; Adding objects to the environment
	• Radiology and wards systems change initiated^	
	• Enteral feeding nurse employed*	
Skills and Beliefs about Capabilities	• Faculty, nurse, and FY1 training with practical elements**	Instruction on how to perform a behavior; Behavioral practice/rehearsal; Increasing skills; Modelling; Social processes of support; Information about health consequences; Credible source
	• E-learning package** (*with video modelling procedure)	
	• Awareness day/week* (also covers social influences)	

*Only implemented as part of the intervention in H2; ** H3 did not have the resource to provide this intervention; ^ = H2 chose not to implement this strategy.

### Feasibility and acceptability

#### Time trend audit

**Figure 1:**
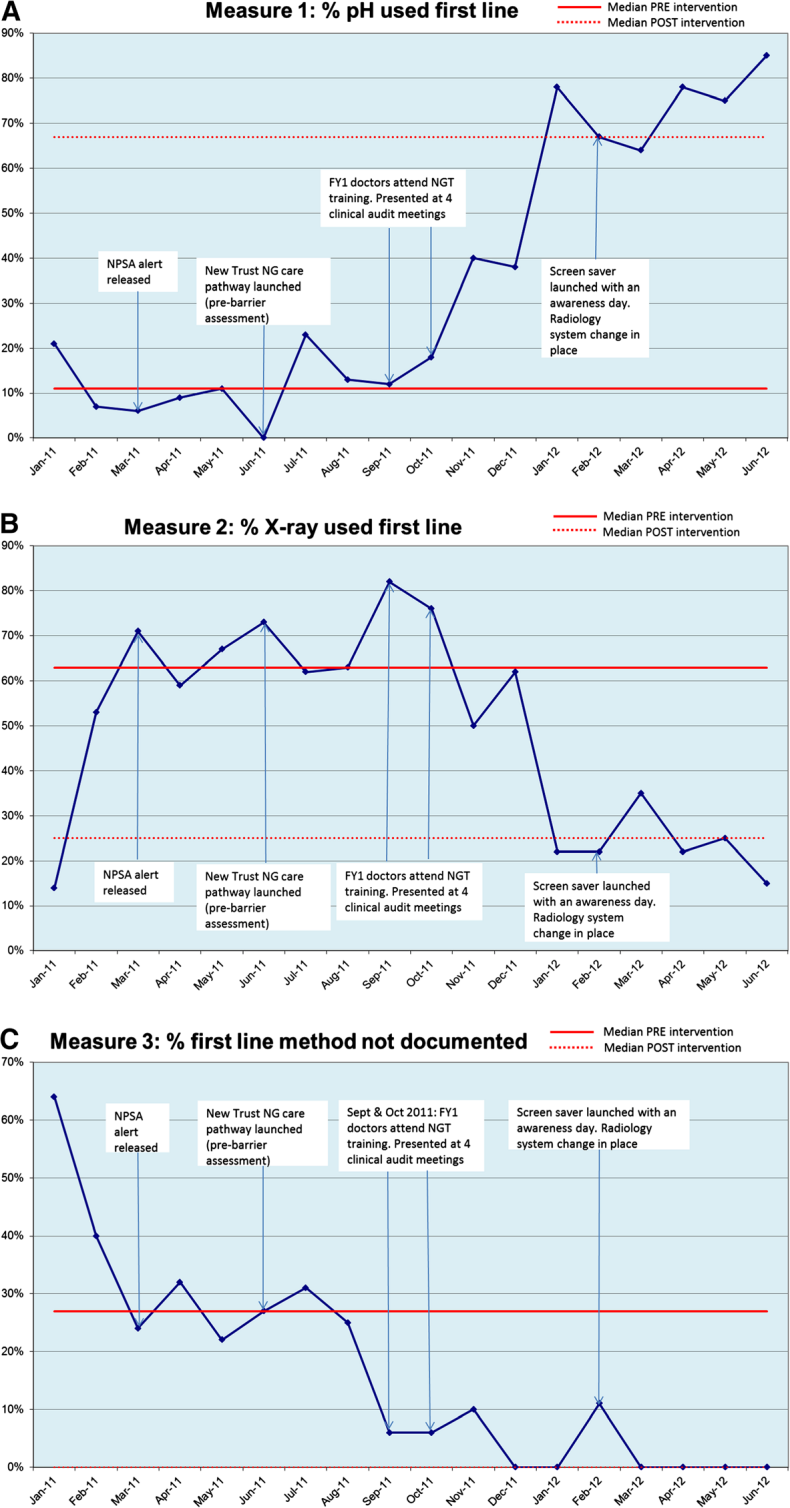
**Time trend for use of pH as first line method used to check tube position.** 1**A**: Use of pH as first line method to check tube position. 1**B**: Use of X-ray as first line method to check tube position. 1**C**: First line method to check tube position not documented.

Trust A audited 301 case notes over 18 months to assess the first line method used to check the position of NG tubes following initial insertion. Fifty seven sets of notes were discarded because patients had received a different tube (*n* = 34) or came into hospital with the ng tube in situ (*n* = 6), the tube was not inserted (*n* = 8), notes were unclear (*n* = 7), or the tube was placed endoscopically (n = 2). The number of included sets of notes per month ranged from nine to 22 (Figure 1A-C). According to Perla, Provost, and Murray [40], Figure 1A-C demonstrate positive 'signals’ in the form of 'shifts’ (six or more consecutive points either all above or all below the median). These positive patterns are supported by the pre-post intervention data (mean percentages for nine months pre-intervention, and nine moths post) which indicates an increase in the use of pH first line from 11% to 60%, a decrease in X-ray from 60% to 37%, and a reduction in the percentage of time practice was not documented (30% to 3%). The Trust interpreted these results as clinically significant.

### Exit interviews

Five telephone interviews were undertaken across the Trusts (H1 = advanced nurse practitioner, and junior doctor, H2 = consultant in care of the elderly, and junior doctor, H3 = gastroenterology consultant) following intervention implementation. Each interview lasted 10 to 20 minutes. Two key themes emerged that directly related to the feasibility and acceptability of this approach, which were: benefits and challenges of the approach; and sustainability (Table 7).

**Table 7:** Exit interview themes, mapped against representative descriptions and example quotes

**Key theme**	**Summary**	**Example quotes**
Benefits	Support provided from the HIEC team	'I guess one of the key things has been the (HIEC team) input; this takes the pressure off the clinicians…without that it wouldn’t have worked so well’ (Consultant, H2).
	The use of behavior change methods throughout the project	'I suppose it’s the behavioral change aspect which was the driving force’ (Consultant, H3).
		'I have had no previous experience in focus groups and that was really where we got most of the ideas for the implementation strategy; it was really useful (Junior doctor, H2).
	The wider impact this work has generated	'It’s got the support of the Deputy Medical Director, it’s really meant that you can have that impact, it’s trust-wide and region-wide as well, whereas normally just a junior doctor doing an audit, it wouldn’t really have that precedence or support or anything (Junior doctor, H2).
Challenges	Having to generate interest and involvement across different areas of the Trust	'Although (through the HIEC team) there’s been a resource to draw on, I do feel overall it would be better to get more hands on deck’ (Consultant, H3).
	Coordinating teams with several and diverse groups	'One of the challenges has been co-ordinating the implementation strategies and actually working with different teams in the hospital like the illustration department, the photographers, the communication experts and the print unit, trying to get everything delivered in a timely manner (Junior doctor, H2).
Sustainability	Spread of information among healthcare professionals	'I’ve also spoken at the regional audit meeting with all the foundation trainees about how being involved in a project where you’ve got frontline staff leading it but with top down support, how you can make a real difference’ (Junior doctor, H2).
	Networks of sharing between hospitals that this work has created	'The knowledge that every other Trust is going through the same issues and wants to improve does create a bit of a network so H1, H2, and H3 are all talking about how to solve this problem’ (Nurse, H1).
	Generated enthusiasm among healthcare professionals for improving patient safety	'For me it’s made me see patient safety in a different aspect like from a much broader base and realising that actually as a junior doctor you really can make a huge difference’ (Junior doctor, H2).

Perceived benefits of the TDFI approach included the support provided from the HIEC team, the use of behavior change methods throughout the project, and the wider impact the work had within organizations, in comparison to previous safety initiatives that participants had been involved in. Challenges included having to generate interest and involvement across different areas of the Trust, and coordinating groups of people involved in intervention development and/or delivery (*e.g.*, medical illustrations, procurement).

Participants described the potential for sustainability of the findings and the approach because: networks had been created and there was greater sharing between professionals and across hospitals, and enthusiasm among healthcare professionals for improving patient safety had been generated.

### Reflective log

The reflective log analysis produced a matrix of the TDFI approach (Table 8) that outlined the implementation steps (the 'what’) against the implementation principles (the 'how’). Context-specific examples of how each implementation principle (see Table 1) was used in stages of the process are provided.

**Table 8:** Matrix of the TDFI approach

**Implementation principles (the 'how’)**	**Behavior change steps (the 'what’)**					
	**Step 1: Form implementation team (IT)**	**Step 2: Identifying the target behavior**	**Step 3: Identifying local barriers (LB)**	**Step 4: Identifying local strategies (LS)**	**Step 5: Implementing local strategies**	**Step 6: Evaluation**
1. The need for management approval and ongoing support	Medical Directors liaised with risk management, quality improvement, frontline staff to determine focus area/gave full support	Management authorized audit to determine target behavior	Management asked to encourage completion of IPSBQ by staff groups involved in target behavior	Management asked to encourage staff to participate in focus groups (FGs)	Management sent LSs by staff in project report and asked for authorization for implementation	Management authorized for post-intervention audit to be undertaken
2. The need for commitment among members of the target group	Recruited IT lead and multi-disciplinary group of staff; expectations clarified to ensure IT members were able to commit to fulfilling their role	IT members encouraged to lead audit to identify target behavior; this involved gaining support/assistance from wards/ departments		Attendance at FGs by staff demonstrated commitment to the improvement of practice	IT members each took responsibility for an element of LSs implementation	
3. Use of boundary spanners	HIEC team acted as boundary spanners by filtering external information into the organizations and linking organizational structure to environmental elements	Fed IT ward staff perceptions about potential target behaviors; IT fed this information both 'up’ and 'down’ their own communication channels; facilitated group to specify exact target behavior	Encouraged IT to distribute IPSBQs to colleagues and encourage completion; fed back findings to IT, clinical governance, junior doctor training, etc.	Facilitated IT to arrange/recruit for FGs; fed information within/between Trusts FGs to gauge LS feasibility; initiated links with Trust areas (*e.g.*, IT; radiology, medical illustrations) for LS implementation	Generated/ facilitated links within/between clinical /non-clinical staff so they could co-produce materials/ resources/ systems for implementation of the LSs; interim report sent to senior management	Will feed results of intervention, experiences, and recommendations for sustainability to IT and senior management in final report
4. Mapping of guidelines onto local problems		Enhanced credibility of guidelines by encouraging IT to audit current practice, and so relating them to local safety issues/ values	Worked with the IT to link key barriers from the IPSBQ to current practice and context (based on audit and discussion)			
5. Adopting the perspective of the target group	Emphasized this not 'performance management’ but aimed to use a 'bottom-up’ approach	Audit data and anecdotal information led IT to make final decision about specific target behavior	Assessing perceived barriers summarized the front-line perspective about the target behavior	Front-line staff generated ideas for LSs, therefore increasing likelihood of adoption	IT members/ward staff were instrumental in the design of SLSs, and/or consulted at key development stages	
6. Acknowledging the complexity of the changing behavior in practice	HIEC team listened to IT members to build a picture about the challenges associated with complying with the alert guidelines	Continuous assessment of audit data/staff discussion to determine main concerns about what was negatively affecting compliance	FGs enabled further understanding about barriers and thus the complexity of the procedure	FGs discussed complex matters; LSs based on experience and understanding of pertinent issues; BCTs addressed deep rooted complexities of LBs	Carefully co-designed and implemented LSs with IT so as not to undermine current staff effort and to highlight justification behind change in practice	
7. A monitoring plan		Audit undertaken; key milestones included post-implementation audit				Post implementation audit /exit interviews underway
8. A flexible approach that is driven by local context	Explained approach aimed to understand/ address perspectives from the 'sharp end of patient care’	Audit strategy based on understanding of wards /departments; target behavior chosen based on Trust resources (*e.g.*, H3 set pH level at 5)	Different methods for IPSBQ data collection (*e.g.*, on-line, paper copy); took into account IT capacity/ other forums to facilitate completion	Timing of FGs arranged to encompass competing priorities for attendees; LSs accounted for existing systems, equipment, resources, staff, etc.	Implementation of LSs aligned with 1) current Trust activities (*e.g.*, clinician rotations, organized training, compliance deadlines, etc.), and 2) capacity of IT to design/implement	
9. Co-production and design to combine theoretical and contextual expertise				Co-developing LSs with multi-disciplinary staff ensured intervention realistic, feasible, simple, and informed by behavior change theory	Co-implementing the SLSs with multi-disciplinary staff meant the intervention was pragmatic, relevant, and theory-based by the operational stage	
10. Incorporation into established structures				SLSs aligned existing equipment, resources, systems; broadcasted practice change via range of mechanisms	Existing Trust services (*e.g.*, medical illustrations, IT) were used to implement LSs	

## Discussion

We have demonstrated a process for developing and implementing theoretically derived, co-designed and context-specific interventions in healthcare organizations. This is the first study to use the TDF to directly inform intervention design in an acute setting, and to outline how specific implementation principles can facilitate the use of the TDF for eliciting behavior change in healthcare settings. This study highlights the importance of local context in identifying barriers and designing and implementing appropriate interventions, and the feasibility and acceptability of the TDFI approach.

There were differences in the key barriers reported among the three organizations, suggesting that local context might have affected perceptions about the challenges faced in complying with a patient safety guideline. We found significant differences between hospitals for three of the domains (knowledge, skills, and emotion); however, there was overlap in the top three barriers identified. For all hospitals, the mean scores for barriers were low, despite poor compliance with recommendations in practice—this could represent a tendency for staff to underestimate the barriers to behavior change, or to respond in a socially desirable way; nonetheless, the relative values between each domain within each organization demonstrate differences in perceptions of barriers, which is important for the purpose of tailoring interventions.

Although some key barriers identified were the same across each Trust (*e.g.*, social influences, environmental context and resources), the detail provided about these barriers in focus groups varied somewhat (*e.g.*, for 'environmental context and resources,’ some staff referred to a lack of adequate documentation, others mentioned how they were unable to easily access pH paper), as did the interventions implemented to overcome these barriers (*e.g.*, to combat the pH paper issue, H2 designed an NG pack that included all the equipment, and H1 worked very closely with procurement to ensure the correct pH strips were available on every ward). The time and resources available to dedicate to this area of improvement in each organization also affected the type of interventions implemented and the degree to which they were developed. For instance, H2 generated an interactive e-learning resource which included video clips of medical staff modelling the entire NG tube procedure—this was achieved due to a dedicated implementation team, who formed working relationships with non-clinical departments (*e.g.*, IT, medical illustrations). In H3, however, IT services were limited and, as such, generating an e-learning package was not possible—instead, to encourage sharing, staff were directed to the H2 e-learning package through posters and screensavers implemented within H3. These examples demonstrate the advantages of adopting the perspective of the target group c.f, [50], using a flexible approach c.f, [39], and incorporating interventions into established structures c.f, [27,51]. Although these implementation principles were used and can be identified in the results of the reflective log analysis (Table 8), using the approach did not come without difficulties, such as resistance to change or a perceived lack of time.

Generally, perceptions of the feasibility and acceptability of the TDFI approach appeared positive; interviewees indicated that the outcomes so far for their organization (*e.g.*, engaging in patient safety work, spreading knowledge) were beneficial. Challenges of this approach included having to gain commitment from staff (*e.g.*, building an implementation team, eliciting contributions from non-clinical departments). However, it is possible that these types of challenges are not exclusive to this approach, but are more common observations of work aiming to produce large scale change within a complex organization [52]. Interviewees expressed appreciation for the HIEC team support throughout the process, and recognized the benefits of holding focus groups to co-design interventions, suggesting that the blend of theoretical support and clinician context expertise [38] worked well. While this feedback suggests that it is feasible and acceptable to use the TDF with healthcare professionals to drive behavior change for patient safety, the support required to ensure teams maintained momentum was resource intensive, and this is highlighted in the results of the reflective log analysis. Irrespective of this point, the positive aspects of this approach for sustainability were evident, including the forming of intra- and inter-organization networks working together on areas of patient safety [53], and the spread of enthusiasm for patient safety work by champions [54].

Collection of time trend data was also feasible. Although we could not perform formal statistical analysis (*i.e.*, time series analysis) given data limitations mentioned earlier, the Trust A audit suggested that 'shifts’ [40] occurred for improved documentation of practice and use of pH first line, and decreased use of X-ray first line, following intervention implementation. Furthermore, the pre-post intervention data indicated clinically significant improvements in practice for all three measurement outcomes. These improvements may be associated with the implementation of the intervention strategies, *e.g.* brief practical training provided for junior doctors (skills; beliefs about capabilities), the presentation of information to senior members of staff at clinical audit meetings (social influences), screensavers and posters (emotion; social influences), and the radiology system change (environmental context and resources; social influences). More formal evaluation methods are needed to establish causation [49,55]; however, these findings indicate potential for co-designing theoretically underpinned interventions to address specific barriers to behavior change for patient safety.

There are two main limitations to our methods. First, participating hospitals were volunteers and therefore may have been more likely to complete the process. However, simply receiving agreement for participation by a medical director did not automatically lead to continued involvement from front line staff. Furthermore, audit data indicated the participating trusts were experiencing issues with guideline implementation that are similar to those faced by others. We also examined a single patient safety alert so, as yet, the extent to which it is possible to use the TDFI approach for other alerts/guidance is unknown. In addition, the number of exit interviews undertaken to understand perceptions of feasibility and acceptability was small (n = 5). Finally, the time trend data was collected only for a single Trust. Pre-post intervention implementation data is being collected for all three Trusts, which we are attempting to compare against retrospectively collected control data.

Second, identifying the successful components of this approach and of the specific interventions on behavior change will also be challenging. For example, at this early stage of development and feasibility testing, it will be difficult to understand the extent of the benefits of using the TDF as part of an approach to implement patient safety guidance in an acute setting, compared with simply providing additional support for implementation. Furthermore, given the range of interventions used to address key barriers, it will be difficult to identify which BCTs have led to change, and whether any change occurred as a result of mediating perceived barriers. Nonetheless, the interventions used have been designed using underlying theory and reported explicitly to enable replication.

Future research should address these limitations by evaluating the TDFI approach within a rigorous randomized control design across more hospitals. There is also scope for using factorial study designs that evaluate combined and separate intervention components (*e.g.*, using the TDF and implementation principles separately), to improve understanding of effects. In addition, it will be important to further refine and test the IPSBQ with larger sample sizes to clarify and improve sensitivity in identifying key barriers to behavior change within organizations, or indeed to establish whether variation genuinely exists as a function of local context.

The outcomes of this work include a framework for the implementation of patient safety guidelines which consists of a) a set of tools to identify context-specific target behaviors to address, barriers to improvement, and theoretically underpinned strategies to overcome barriers, alongside b) a set of implementation principles to guide use of these tools with organizations. The TDFI approach and the associated resources may be of use in other healthcare organizations and to guideline implementation teams and policy makers, especially if the post-intervention data from the three Trusts indicate changes in behavior in comparison to control sites.

## Conclusion

It is feasible and acceptable to combine theory-driven and co-design approaches in the development of strategies to support the implementation of an evidence-based patient safety guideline. The impact of local context and value of local expertise should not be under-estimated. Future work should replicate or adapt theory-driven, co-designed interventions and evaluate their effects within rigorous designs.

## Abbreviations

IPSBQ: Influences on patient safety behaviors questionnaire; TDF: Theoretical sramework; TDFI approach: Theoretical domains framework implementation approach; NPSA: National patient safety agency; NG: Nasogastric; H1, H2, H3: Hospital 1, Hospital 2, Hospital 3; BCT: Behavior change technique.

## Competing interests

The authors declare that they have no competing interests.

## Authors’ contributions

NT led the design and coordination of the study, performed the statistical analysis, and led the writing process. RL participated in the design of the study, and helped to draft the manuscript. BS participated in the design of the study, and helped to draft the manuscript. RF helped to draft the manuscript. All authors read an approved the final manuscript.

## Authors’ information

NT and RL have previously worked on projects that involve using the TDF framework to identify barriers and design interventions using theoretically underpinned behavior change techniques to design tailored interventions to address key barriers for a range of health behaviors. RL conceived the original idea for using the TDF to implement NPSA guidelines. NT developed the TDFI approach. BS is an organizational psychologist who has drawn upon her knowledge and experience of implementation science to contribute to this work. RF is a general practitioner and implementation researcher, and is also the Deputy Editor of Implementation Science; all decisions on this manuscript were made by another editor.

## Supplementary Material

Additional file 1 Summary of the TDF implementation approach and outcomes. Click here for file

Additional file 2 Focus group interview schedule. Click here for file

Additional file 3 Exit interview schedule. Click here for file

## References

[B1] Andrews LB Stocking C Krizek T Gottlieb L Krizek C Vargish T Siegler M An alternative strategy for studying adverse events in medical care Lancet 1997 349 309 313 10.1016/S0140-6736(96)08268-2 9024373

[B2] Brennan TA Leape LL Laird NM Hebert L Localio AR Lawthers AG Newhouse JP Weiler PC Hiatt HH Incidence of adverse events and negligence in hospitalized patients: results of the harvard medical practice study I (reprinted from New england journal of medicine, vol 324, pg 370–7, 1991) Qual Saf Health Care 2004 13 145 151 10.1136/qshc.2002.003822 15069223 PMC1743811

[B3] Vincent C Stanhope N Crowley‒Murphy M Reasons for not reporting adverse incidents: an empirical study J Evaluat Clin Prac 1999 5 13 21 10.1046/j.1365-2753.1999.00147.x 10468380

[B4] Reason J Human error: models and management Br Med J 2000 320 768 770 10.1136/bmj.320.7237.768 10720363 PMC1117770

[B5] Lawton RJ McEachan RRC Giles SJ Sirriyeh R Watt IS Wright J Development of an evidence-based framework of factors contributing to patient safety incidents in hospital settings: a systematic review BMJ Quality and Safety 2012 21 369 380 OnlineFirst 10.1136/bmjqs-2011-000443 22421911 PMC3332004

[B6] Asch SM Kerr EA Keesey J Adams JL Setodji CM Malik S McGlynn EA Who is at greatest risk for receiving poor-quality health care? N Engl J Med 2006 354 1147 1156 10.1056/NEJMsa044464 16540615

[B7] Runciman WB Hunt TD Hannaford NA Hibbert PD Westbrook J Coiera EW Day RO Hindmarsh DM McGlynn EA Braithwaite J CareTrack: assessing the appropriateness of healthcare delivery in australia Med J Aust 2012 197 100 105 10.5694/mja12.10510 22794056

[B8] Grimshaw JM Thomas RE MacLennan G Fraser C Ramsay CR Vale L Whitty P Eccles MP Matowe L Shirran L Effectiveness and efficiency of guideline dissemination and implementation strategies Health Technol Assess 2004 8 1 10.3310/hta8060 14960256

[B9] Shojania KG Grimshaw JM Evidence-based quality improvement: the state of the science Health Aff 2005 24 138 150 10.1377/hlthaff.24.1.138 15647225

[B10] Grol R Personal paper. Beliefs and evidence in changing clinical practice BMJ 1997 315 418 10.1136/bmj.315.7105.418 9277610 PMC2127297

[B11] Davies P Walker AE Grimshaw JM A systematic review of the use of theory in the design of guideline dissemination and implementation strategies and interpretation of the results of rigorous evaluations Implement Sci 2010 5 doi:10.1186/1748-5908-5-14 10.1186/1748-5908-5-14 PMC2832624 20181130

[B12] Grol R Grimshaw J From best evidence to best practice: effective implementation of change in patients’ care Lancet 2003 362 1225 1230 10.1016/S0140-6736(03)14546-1 14568747

[B13] Vincent C Patient Safety 2011 Chichester, West Sussex, UK: Wiley-Blackwell

[B14] Hawe P Shiell A Riley T Gold L Methods for exploring implementation variation and local context within a cluster randomised community intervention trial J Epidemiol Community Health 2004 58 788 793 10.1136/jech.2003.014415 15310806 PMC1732876

[B15] Øvretveit JC Shekelle PG Dy SM McDonald KM Hempel S Pronovost P Rubenstein L Taylor SL Foy R Wachter RM How does context affect interventions to improve patient safety? an assessment of evidence from studies of five patient safety practices and proposals for research BMJ Quality & Safety 2011 20 604 610 10.1136/bmjqs.2010.047035 21493589

[B16] Bosk CL Dixon-Woods M Goeschel CA Pronovost PJ Reality check for checklists Lancet 2009 374 444 445 10.1016/S0140-6736(09)61440-9 19681190

[B17] Leistikow IP Kalkman CJ Bruijn H Why patient safety is such a tough nut to crack BMJ 2011 34 188 190 10.1136/bmj.d3447 21693533

[B18] Grol R Bosch MC Hulscher MEJL Eccles MP Wensing M Planning and studying improvement in patient care: the use of theoretical perspectives Milbank Q 2007 85 93 138 10.1111/j.1468-0009.2007.00478.x 17319808 PMC2690312

[B19] Michie S Johnston M Abraham C Lawton R Parker D Walker A Making psychological theory useful for implementing evidence based practice: a consensus approach Qual Safety Health Care 2005 14 26 33 10.1136/qshc.2004.011155 PMC1743963 15692000

[B20] Dyson J Lawton R Jackson C Cheater F Does the use of a theoretical approach tell us more about hand hygiene behaviour? the barriers and levers to hand hygiene J Infect Prev 2011 12 17 24 10.1177/1757177410384300

[B21] Francis JJ Stockton C Eccles MP Johnston M Cuthbertson BH Grimshaw JM Hyde C Tinmouth A Stanworth SJ Evidence‒based selection of theories for designing behaviour change interventions: using methods based on theoretical construct domains to understand clinicians’ blood transfusion behaviour Br J Health Psychol 2009 14 625 646 10.1348/135910708X397025 19159506

[B22] McCluskey A Middleton S Delivering an evidence-based outdoor journey intervention to people with stroke: barriers and enablers experienced by community rehabilitation teams BMC Health Serv Res 2010 10 18 10.1186/1472-6963-10-18 20082725 PMC2821384

[B23] Amemori M Korhonen T Kinnunen T Michie S Murtomaa H Enhancing implementation of tobacco use prevention and cessation counselling guideline among dental providers: a cluster randomised controlled trial Implement Sci 2011 6 13 10.1186/1748-5908-6-13 21320312 PMC3055178

[B24] Patey AM Islam R Francis JJ Bryson GL Grimshaw JM Anesthesiologists’ And surgeons’ perceptions about routine pre-operative testing in low-risk patients: application of the theoretical domains framework (TDF) to identify factors that influence physicians’ decisions to order pre-operative tests Implement Sci 2012 7 doi: 10.1186/1748-5908-7-52 10.1186/1748-5908-7-52 PMC3522997 22682612

[B25] Cane J O’Connor D Michie S Validation of the theoretical domains framework for use in behaviour change and implementation research Implement Sci 2012 7 doi: 10.1186/1748-5908-7-37 10.1186/1748-5908-7-37 PMC3483008 22530986

[B26] Taylor N Lawton R Conner MC Development and initial validation of the determinants of physical activity questionnaire Inter JBehav NutrPhysic Act 2013 10 doi:10.1186/1479-5868-10-74 10.1186/1479-5868-10-74 PMC3684519 23758912

[B27] Greenhalgh T Robert G Macfarlane F Bate P Kyriakidou O Diffusion of innovations in service organizations: systematic review and recommendations Milbank Q 2004 82 581 629 10.1111/j.0887-378X.2004.00325.x 15595944 PMC2690184

[B28] Grol R Wensing M Eccles M Improving patient care: the implementation of change in clinical practice 2005 Heinemann Edinburgh: Elsevier Butterworth

[B29] Rogers EM Diffusion of innovations 1995 New York: Free Pr

[B30] Kimberly JR Evanisko MJ Organizational innovation: the influence of individual, organizational, and contextual factors on hospital adoption of technological and administrative innovations Acad Manag J 1981 34 3 689 713 10253688

[B31] Aldrich H Herker D Boundry spanning roles and organizational structure Acad Manag Rev 1977 2 217 230

[B32] Burgers JS Grol RPTM Zaat JOM Spies TH van der Bij AK Mokkink HGA Characteristics of effective clinical guidelines for general practice Br J Gen Pract 2003 53 15 12569898 PMC1314503

[B33] Davis DA Taylor-Vaisey A Translating guidelines into practice. A systematic review of theoretic concepts, practical experience and research evidence in the adoption of clinical practice guidelines Can Med Assoc J 1997 157 408 416 9275952 PMC1227916

[B34] Foy R MacLennan G Grimshaw J Penney G Campbell M Grol R Attributes of clinical recommendations that influence change in practice following audit and feedback J Clin Epidemiol 2002 55 717 722 10.1016/S0895-4356(02)00403-1 12160920

[B35] Graham ID Logan J Harrison MB Straus SE Tetroe J Caswell W Robinson N Lost in knowledge translation: time for a map? J Contin Educ Health Prof 2006 26 13 24 10.1002/chp.47 16557505

[B36] Grol R Dalhuijsen J Thomas S Rutten G Mokkink H Attributes of clinical guidelines that influence use of guidelines in general practice: observational study BMJ 1998 317 858 861 9748183 10.1136/bmj.317.7162.858 PMC31096

[B37] Pronovost PJ Berenholtz SM Needham DM Translating evidence into practice: a model for large scale knowledge translation BMJ 2008 337 963 965 10.1136/bmj.a963 18838424

[B38] Greenhalgh T Robert G Macfarlane F Bate P Kyriakidou O Peacock R Storylines of research in diffusion of innovation: a meta-narrative approach to systematic review Soc Sci Med 2005 61 417 430 10.1016/j.socscimed.2004.12.001 15893056

[B39] Lewis R Fletcher M Implementing a national strategy for patient safety: lessons from the national health service in england Qual Saf Health Care 2005 14 135 139 10.1136/qshc.2004.011882 15805460 PMC1743990

[B40] Perla RJ Provost LP Murray SK The run chart: a simple analytical tool for learning from variation in healthcare processes BMJ Qual and Safety 2011 20 46 51 10.1136/bmjqs.2009.037895 21228075

[B41] French SD Green SE O’Connor DA McKenzie JE Francis JJ Michie S Buchbinder R Schattner P Spike N Grimshaw JM Developing theory-informed behaviour change interventions to implement evidence into practice: a systematic approach using the theoretical domains framework Implement Sci 2012 7 38 10.1186/1748-5908-7-38 22531013 PMC3443064

[B42] NPSA Patient safety alert: reducing the harm caused by misplaced nasogastric feeding tubes 2011 London, UK: NHS National Patient Safety Agency

[B43] Taylor N Parveen S Robins V Slater B Lawton R Development and initial validation of the influences on patient safety behaviours questionnaire Implement Sci 2013 8 doi:10.1186/1748-5908-8-81 10.1186/1748-5908-8-81 PMC3846501 23895628

[B44] Michie S Johnston M Francis J Hardeman W Eccles M From theory to intervention: mapping theoretically derived behavioural determinants to behaviour change techniques Appl Psychol 2008 57 660 680 10.1111/j.1464-0597.2008.00341.x

[B45] Abraham C Michie S A taxonomy of behavior change techniques used in interventions Health Psychol 2008 27 379 18624603 10.1037/0278-6133.27.3.379

[B46] Craig P Dieppe P Macintyre S Michie S Nazareth I Petticrew M Developing and evaluating complex interventions: the new medical research council guidance BMJ 2008 337 a1655 a1655 10.1136/bmj.a1655 18824488 PMC2769032

[B47] Crabtree B Doing Qualitative Research 1999 Newbury Park, CA: Sage

[B48] Michie S Abraham C Eccles MP Francis JJ Hardeman W Johnston M Strengthening evaluation and implementation by specifying components of behaviour change interventions: a study protocol Implement Sci 2011 6 doi:10.1186/1748-5908-6-10 10.1186/1748-5908-6-10 PMC3041694 21299860

[B49] Ramsay CR Matowe L Grilli R Grimshaw JM Thomas R Interrupted time series designs in health technology assessment: lessons from two systematic reviews of behaviour change strategies Inter J Technol Assess Health Care 2003 19 613 623 10.1017/s0266462303000576 15095767

[B50] Grol R Wensing M Grol R, Wensing M, Eccles M Effective Implementation: A Model Improving Patient Care; the Implementation of Change in Clinical Practice 2005 Oxford: Elsevier 41 58

[B51] Damanpour F Organizational innovation: a meta-analysis of effects of determinants and moderators Acad Manage J 1991 34 3 555 590 10.2307/256406

[B52] Weiner B A theory of organisational readiness for change Implement Sci 2009 4 doi: 10.1186/1748-5908-4-67 10.1186/1748-5908-4-67 PMC2770024 19840381

[B53] Braithwaite J Runciman WB Merry F Towards safer, better healthcare: harnessing the natural properties of complex sociotechnical systems BMJ Qual and Safety 2009 18 37 44 10.1136/qshc.2007.023317 PMC2629006 19204130

[B54] Soo S Berta W Baker R Role of champions in implementing patient safety practice change Healthc Q 2009 12 123 128 10.12927/hcq.2009.20979 19667789

[B55] Eccles M Grimshaw J Campbell M Ramsay CR Research designs for studies evaluating the effectiveness of change and improvement strategies Qual and Safety in Healthcare 2003 12 47 52 10.1136/qhc.12.1.47 PMC1743658 12571345

