# Supplementary material for: The demonstration of a theory-based approach to the design of localized patient safety interventions
Source: Implement Sci. 2013 Oct 16;8:123. doi: 10.1186/1748-5908-8-123 (PMC3854455; doi:10.1186/1748-5908-8-123)
Supplement: Additional file 1 — Summary of the TDF implementation approach and outcomes. [file 1748-5908-8-123-S1.pdf]

Additional File 1: Summary of implementation steps and outcomes

Step	Description	Outcome
1: Forming an implementation team.	• Implementation teams formed to build relationships, establish project objectives, ensure coordination, and plan outcome measurement.<br>• Teams presented with the approach, agreed to participate on this basis, and met around once every six weeks.	• The formation and operation of implementation teams, and staff numbers and type belonging to each team varied by hospital.<br>• H1 team: led by and advanced nutrition support nurse and consisted of three junior doctors, a nutrition support nurse, and a dietician.<br>• H2 team: led by a consultant gastroenterologist, and consisted of one junior doctor, a dietician, a matron, and two registrars.<br>• H3 team: led by an Elderly medicine Consultant and consisted of a consultant gastroenterologist, two registrars, four junior doctors, a nurse, and a medical student
2: Defining a locally relevant target behaviour.	• Implementation teams discussed the alert in relation to local practice to inform the decision about areas of the hospital to audit, and develop initial ideas about which target behaviour to focus on.<br>• Teams supported to audit current NG tubes practice using co-developed audit tools to confirm target behaviour.	• Implementation team and ward staff discussions in each hospital indicated that the main area of concern they had with complying with the alert was the first line method used to check the position of NG tubes.<br>• Baseline audits confirmed this as an issue (Table 3) – taking an average across all three hospitals, using pH as the first line method to check tube position was undertaken only 14% of the time, whereas X-ray was being used 55% of the time.<br>• Each hospital decided that the target behaviour for change would be for staff to check pH first line.
3: Understanding the barriers to performing the desired behaviour.	*IPSBQ*<br>• The IPSBQ was distributed to all staff that had some involvement in the target behaviour.<br>• Mean domain scores were calculated for each hospital to establish key barriers to behaviour change.	*IPSBQ*<br>• The strongest barrier to performing the target behaviour (checking pH first line) across H1 and H2 was 'social influences' (the influence of others on the behaviour), and for H3 was 'skills (having the necessary training and skills to perform the behaviour). There were differences across

Step	Description	Outcome
		sites with regards to other reported barriers.
	Focus groups<br>• Following analysis of IPSBQ data, focus groups were held at each hospital with multi-disciplinary staff groups from a range of wards and departments to gain a detailed and contextual understanding of key barriers.	*Focus groups*<br>• H1: two focus groups, 10 members of staff (four junior doctors, three nurses, one sister, one nutrition support nurse, and one operation department practitioner); H2: four focus groups, 26 staff members (eight junior doctors, three registrars, six nurses, two dieticians, one physiotherapist, and one consultant); H3: two focus groups, eight staff members (three nurses, one senior dietician, one consultant, and three junior doctors).<br>• Responses were provided from staff operating at various levels regarding the key barriers in each Trust, examples of which are presented in Table 5 and mapped against TDF domains.
4: Devising intervention strategies to address identified barriers.	• Staff in focus groups discussed ideas for intervention strategies that they envisaged would be effective in addressing the most prominent barriers and achieving the target behaviour.<br>• Generation of the ideas by each group was guided by the project team’s knowledge of the current behaviour change literature [30-32] (i.e., the use of specific behaviour change techniques for addressing key barriers).<br>• The key barriers emerging from the focus groups were cross referenced with those identified by the IPSBQ.<br>• Suggested intervention strategies were matched to the specific barriers identified, then mapped against BCTs [31, 34].	• Examples of intervention suggestions which have been matched to quotes representing barriers from specific TDF domains are presented in Table 5.<br>• To provide transparency about the intervention development process, mapping of BCTs to barriers was made explicit. For example, in instances where strategies were suggested by participants which represented BCTs which had not been previously matched to specific barriers in the literature, it was important to use them if they were perceived by the majority as a valuable and feasible solution to overcoming a barrier.

Step	Description	Outcome
5: Intervention implementation.	• Reports produced for senior management in each hospital, which included an explanation of the approach taken to identify the target behaviour, predominant barriers to performing the target behaviour, and interventions to address the barriers.<br>• Senior management asked to approve the recommended interventions, and provide any resources necessary to support implementation within the Trust.<br>• Once the report was returned with authorisation of the interventions, teams were supported to implement the strategies in each Trust.	• Strategies authorised, developed, and implemented across the three Trusts are presented in Table 6.<br>• Authorisation was provided for the majority of strategies; senior management provided reasons for rejecting a suggested intervention.
6: Evaluation	• Organisations were supported to carry out pPost-intervention audits to determine the impact of the interventions (since the initial audit undertaken in Step 2) on the performance of the target behaviour.	• Post-intervention implementation audits have been undertaken in each Trust and will be reported in a forthcoming paper.
