# Supplementary material for: The demonstration of a theory-based approach to the design of localized patient safety interventions
Source: Implement Sci. 2013 Oct 16;8:123. doi: 10.1186/1748-5908-8-123 (PMC3854455; doi:10.1186/1748-5908-8-123)
Supplement: Additional file 2 — Focus group interview schedule. [file 1748-5908-8-123-S2.pdf]

FOCUS GROUP TOPIC GUIDE EXAMPLE

Practitioner barriers to checking the position of NG tubes

Introduction and informed consent: 10mins

- Welcome
- Introduce topic: practitioner barriers to checking the position of NG tubes
- State purpose of focus groups (e.g. use to develop intervention etc)
- Discussion take about 40-60 minutes, ask about your views in relation to checking the position of nasogastric tubes
- Will be recording the discussion
- Assure of confidentiality and anonymity
- Voluntary participation and right to withdraw without giving a reason

Introductions and info about practitioners:

- Brief introduction – name, profession, area of work, years of experience

Define problem:

- Feeding through misplaced ng tubes
- Briefly explain the common approach to patient safety alerts, the behaviour change gap and our approach to break down/fix the problem
- Define problem behaviour: sending for X-ray as first line method (rather than checking the pH of the aspirate, or despite obtaining aspirate < 5.5)

Barriers to checking the pH of the aspirate and relying on the result in order to commence feeding

1. Present list of possible barriers **(exercise 1)**
   a. Talk through each type of barrier in the context of ng tubes problem behaviour
   b. Then ask to rate the top 4-5 they believe exist within the trust
2. Present preliminary results of questionnaire
   a. Determinant scores were ranked (based on number of times rated as a strong barrier by individuals) to assess different aspects of implementation difficulties. Mean scores for each determinant were also computed
   b. The determinant 'social influences' was ranked as a strong barrier by more participants than any other determinant area, which was followed by 'skills', and 'environmental context and resources'
   c. High scores (4/5) represent barriers – no areas produced a mean score > 3, suggesting staff do not perceive barriers to ensuring patients are not sent for an x-ray unless it is impossible to obtain aspirate or pH level is > 5.0
   d. However, audit data suggests otherwise (most patients are sent for an x-ray)
   e. Discuss similarities and differences
3. Arrive at a general consensus and decide which areas they think we should target
   a. Create definitive list of top 4-5 barriers
4. Devise set of strategies to tackle barriers **(exercise 2)**
   a. Take in list of taxonomy techniques mapped to determinant areas **(discuss with attendees where possible)**

Exercise 1: Practitioner barriers to checking the position of NG tubes

Please rate the following areas in terms of the largest (1) and smallest (11) barriers to ensuring that patients are not sent for an x-ray unless it is impossible to obtain aspirate or the pH level is > 5.0

Knowledge (about checking pH level of the aspirate as the first line method to confirm tube position – *as opposed to sending for an X-ray as first line method*)

- Staff unaware that they should do this
- Do not know why it is important
- Do not agree with the guidelines
- Don't know where to find information to help

____/11

Skills (that enable staff to check pH level of aspirate as the first line method to confirm tube position – *as opposed to sending for an X-ray as first line method*)

- Staff do not have the necessary skills
- Training isn't offered regularly enough
- Training is not adequate

____/11

Professional identity

- Staff do not think it's an integral part of their duty of care
- Do not think it's their responsibility
- Are not clear about what their role should be in the process

____/11

Beliefs about capabilities

- Staff are not confident about the aspirate method
- Do not find it easy to use the aspirate method
- Have previously encountered problems when using the aspirate method

____/11

Beliefs about consequences

- Staff don't think it matters too much if they send for an x-ray first line or if even if they get an aspirate within the necessary range
- Don't think it will be a bad thing if the patient is sent for an x-ray first line
- Staff think the costs outweigh the benefits of sending for aspirate first line

____/11

Motivation and goals

- Staff generally intend to send for an x-ray first line or even if aspirate in specified range
- Other guidelines conflict with the ng tubes guidelines about checking the aspirate as first line
- Other priorities get in the way

____/11

Cognitive processes, memory and decision making

- Staff think it's justified to send for x-ray first line or even if ph within the range
- Staff forget to check the aspirate first line
- Staff do not usually check the aspirate first line

____/11

Environmental context and resources

____/11

- The necessary resources are not available
- Verbal and written communication is not clear enough between staff
- There is not a good enough system in place to ensure aspirate is used as first line

Social Influences

____/11

- Other staff do not encourage testing the aspirate first line
- Most staff don't test the aspirate first line (or generally tend to send for an x-ray)
- Superiors do not express that they would like staff to test the aspirate first line

Emotion

____/11

- Staff are anxious about having to trust a pH level
- Staff worry about trusting the ph level
- Staff feel frustrated about having to test the ph level

Behavioural regulation

____/11

- Staff do not plan how they will ensure they check ph level as first line
- Staff get plans mixed up regarding checking the ph level
- Things are too unpredictable for staff to make plans to check ph level first time

Any barriers that we have missed...?

Exercise 2: Top 5 barriers, intervention strategies, and coded techniques

Top 5 barriers	Suggested intervention strategies	Coded techniques
