# Supplementary material for: The demonstration of a theory-based approach to the design of localized patient safety interventions
Source: Implement Sci. 2013 Oct 16;8:123. doi: 10.1186/1748-5908-8-123 (PMC3854455; doi:10.1186/1748-5908-8-123)
Supplement: Additional file 3 — Exit interview schedule. [file 1748-5908-8-123-S3.pdf]

INTERVIEW TOPIC GUIDE

Practitioner experiences of working with the HIEC to implement patient safety alerts

Introduction and informed consent

- Welcome
- Introduce topic: practitioner experiences of working with the HIEC to use a behaviour change approach to implementing patient safety alerts
- State purpose of interview (e.g. to understand practitioner experiences, challenges faced, new learning, new relationships, changed practice, suggested improvements, etc. in order to inform the development of materials to support future projects)
- Discussion take about 20 minutes, ask about your experiences
- Will be recording the discussion
- Assure of confidentiality and anonymity
- Voluntary participation and right to withdraw without giving a reason

Introductions and info about practitioners:

- Brief introduction – name, profession, area of work, years of experience, role in the HIEC work (e.g., steering group lead, undertook audits, organising focus groups, developed interventions, etc.)

Question list

1) Could you tell me about your experiences of being involved in this project including those things that you felt worked well and those things that were more difficult?

2) In what way was this approach different to how you would normally make changes or implement safety policy?

3) What impact has being involved in this project had on:
    - You
    - The group
    - Your relationships with people in the Trust
    - Patients

4) What aspects of the support you received from the HIEC team were:
    - Most useful
    - Least useful

5) Have you shared your ideas or experiences of this project with colleagues or others within or outside this Trust? Please tell us about this.

6) Finally, how will you use what you have learnt in this project in your future work?
